# Soma-to-Germline Transmission of RNA in Mice Xenografted with Human Tumour Cells: Possible Transport by Exosomes

**DOI:** 10.1371/journal.pone.0101629

**Published:** 2014-07-03

**Authors:** Cristina Cossetti, Luana Lugini, Letizia Astrologo, Isabella Saggio, Stefano Fais, Corrado Spadafora

**Affiliations:** 1 SBGSA, Istituto Superiore di Sanità, Rome, Italy; 2 Unit of Antitumor Drugs, Department of Therapeutic Research and Medicines Evaluation, Istituto Superiore di Sanità, Rome, Italy; 3 Department of Biology and Biotechnology “Charles Darwin”, Sapienza University, Rome, Italy; Gustave Roussy, France

## Abstract

Mendelian laws provide the universal founding paradigm for the mechanism of genetic inheritance through which characters are segregated and assorted. In recent years, however, parallel with the rapid growth of epigenetic studies, cases of inheritance deviating from Mendelian patterns have emerged. Growing studies underscore phenotypic variations and increased risk of pathologies that are transgenerationally inherited in a non-Mendelian fashion in the absence of any classically identifiable mutation or predisposing genetic lesion in the genome of individuals who develop the disease. Non-Mendelian inheritance is most often transmitted through the germline in consequence of primary events occurring in somatic cells, implying soma-to-germline transmission of information. While studies of sperm cells suggest that epigenetic variations can potentially underlie phenotypic alterations across generations, no instance of transmission of DNA- or RNA-mediated information from somatic to germ cells has been reported as yet. To address these issues, we have now generated a mouse model xenografted with human melanoma cells stably expressing EGFP-encoding plasmid. We find that EGFP RNA is released from the xenografted human cells into the bloodstream and eventually in spermatozoa of the mice. Tumor-released EGFP RNA is associated with an extracellular fraction processed for exosome purification and expressing exosomal markers, in all steps of the process, from the xenografted cancer cells to the spermatozoa of the recipient animals, strongly suggesting that exosomes are the carriers of a flow of information from somatic cells to gametes. Together, these results indicate that somatic RNA is transferred to sperm cells, which can therefore act as the final recipients of somatic cell-derived information.

## Introduction

In recent years epigenetic studies are growingly disclosing unexpected features of genome function and structure, gradually leading to reconsider the established view that Mendelian laws are the unique paradigm for the mechanism of inheritance of genetic information from one generation to the next. A growing body of data now supports the view that genetic information can be transmitted via non-Mendelian transgenerational inheritance, a phenomenon in which traits unlinked to chromosomal genes are transmitted to the progeny, generating persistent phenotypes [1]. Different routes of transmission of epigenetic information are possible, which may either involve the germline or be germline-independent. Under particular conditions, germline-independent transmission of epigenetic alterations is caused by direct environmental exposure that influences the offspring phenotypes – e.g., effect of the mother’s diet through the placenta, or transfer of the mother’s acquired immunity through mammary gland secretions. In other cases, when the environmental exposure effects cause phenotypic variations that, intriguingly, are transmitted through generations in the non-exposed progeny, the transgenerational inheritance is recognized as germline-dependent [2]–[4]. A variety of pathologies with onset in adult life display non-Mendelian transgenerational inheritance, including obesity, type-2 diabetes, cardiovascular diseases [5], [6], some tumor types [7] and schizophrenia [8]. No classical mutations or predisposing genetic lesions are identified in the genome of individuals that develop these diseases although transgenerational inheritance of epigenetic states, i.e. variation in patterns of DNA methylation and of DNA-binding histone proteins, has been reported [9], [10]. Growing evidence also points to RNA as a carrier of epigenetic information that can be transgenerationally transmitted: RNA is pervasively transcribed throughout the genome [11], cell-free RNAs circulate in blood [12] and, furthermore, complex RNA populations [4] accumulate in mature spermatozoa nuclei [13] and hence can be delivered to oocytes at fertilization [14]. Studies with murine models actually suggest that RNA is the transgenerational determinant of inheritable epigenetic variations and that spermatozoal RNA can carry and deliver information that cause phenotypic variations in the progeny. Well-documented evidence indeed implicate sperm miRNAs in transgenerational epigenetic inheritance of expression at the *Kit* locus, which is crucial in mouse development [15], and of the Cdk9 transcription factor, which causes a transmissible epigenetic pathological hypertrophy of the heart [16]. In both cases, the sperm RNA population of phenotypically affected males is characterized by the accumulation of non-polyadenylated RNA molecules of abnormal size which, when microinjected in fertilized oocytes, are sufficient to induce mutant phenotypes in the newborn mice. The latter observations suggest that spermatozoal RNA molecules can act as epigenetic determinants with functional roles in embryonic development [17]. The origin of these aberrant RNAs is not known, but does not seem to reside within the germline. An intriguing hypothesis suggested by these results is that an RNA-mediated transfer of information may occur between somatic and germ cells, and that spermatozoa might be the final recipients of somatic RNA populations.

To test this hypothesis, we have now devised an experimental strategy using murine models xenografted with human tumor cells, which are known to act as a major releasing source of circulating nucleic acids [12] and such as exosomes [18]–[20]; the latter are released in the circulating blood and are carriers of both RNA and DNA molecules [18]–[21]. We have generated a human A-375 melanoma-derived EGFP-expressing cell line and have used it for xenografts into nude mice. We find that EGFP-specific RNA is indeed released in the circulating blood of the animals, associated with tumor cell-derived extracellular preparations processed for exosome purification, and is eventually delivered to and stored in mature spermatozoa. To our knowledge, this is the first evidence for the passage of RNA molecules from the soma to mature germ cells.

## Results

### EGFP RNA is found in the circulating blood from nude mice xenografted with human A-375 melanoma-derived EGFP-expressing cells

**Figure 1:**
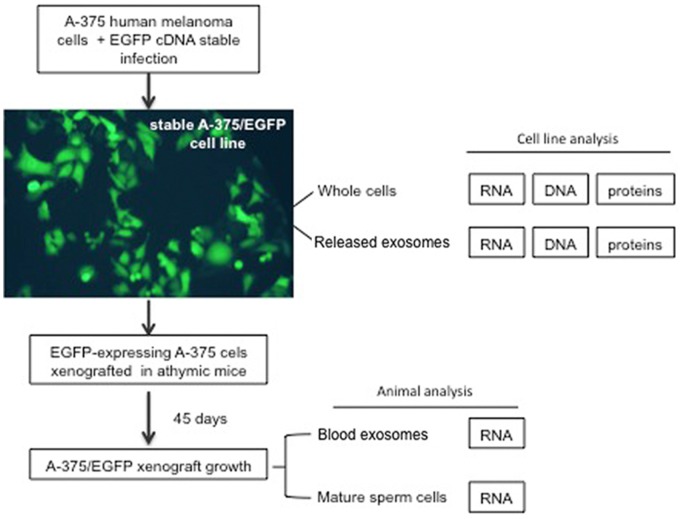
Outline of the general procedure used for the stepwise detection of EGFP expression from tumor to sperm cells. An A-375 melanoma derivative cell line stably expressing the EGFP reporter gene was obtained by infecting with an engineered letiviral vector. EGFP RNA, DNA and proteins were detected both in whole A-375 cells and in A-375-released exosomes. Cells were then xenografted in nude mice, 45 days after inoculation the animals were sacrificed and both blood-released exosomes and epidydimal spermatozoa were analyzed for EGFP-containing RNA.

**Figure 2:**
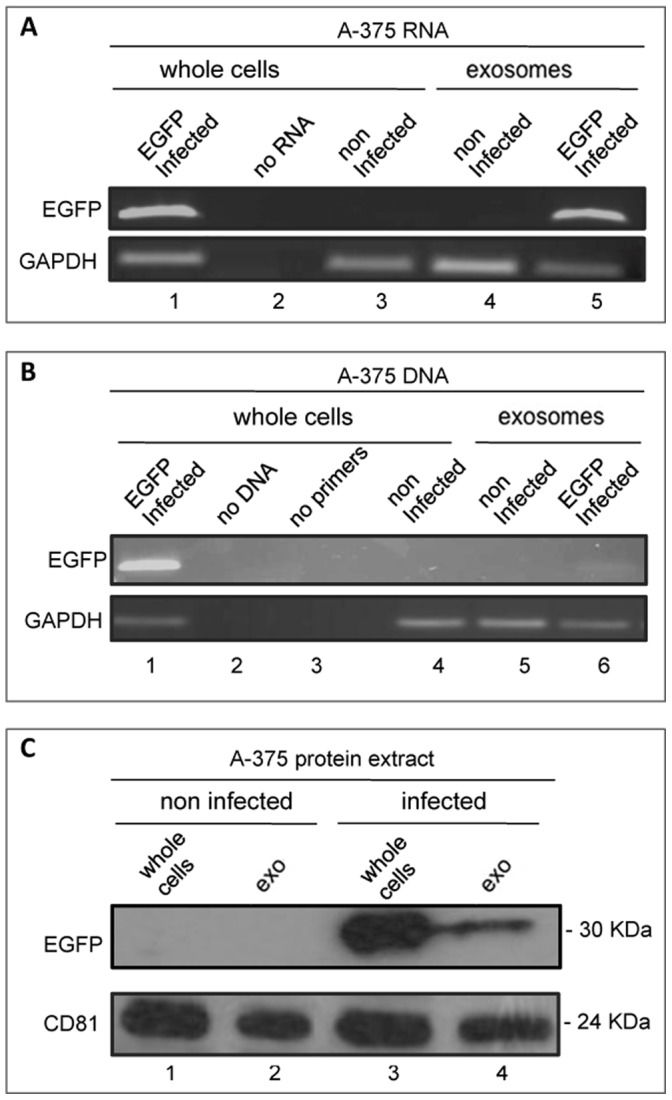
Characterization of EGFP-expressing A-375 cells. **A**: EGFP-specific RT-PCR amplification from RNA extracted from whole A-375 cells, either EGFP-infected (lane 1) or non-infected (lane 3), and from the extracellular exosome-containing fraction of infected (lane 5) and non-infected (lane 4) A-375 cells. In lane 2 no RNA was added to the amplification mix. GAPDH was used as standard control. **B**: PCR amplification of DNA extracted from whole A-375 cells, EGFP-infected (lane 1) and non-infected (lane 4), and from the extracellular exosomal fraction from infected (lane 6) or non-infected (lane 5) A-375; no DNA- and no primer-reactions were loaded for control in lanes 2 and 3, respectively. **C**: Western immunoblotting analysis of protein extracts from: non-infected A-375 cells (lane 1) and exosomal fraction (lane 2), and from infected A-375 cells (lane 3) and their exosomal fraction (lane 4). CD81 was used as a marker of exosomes.

The purpose of this work was to investigate whether mature spermatozoa can carry RNAs of somatic origin that can potentially cause transgenerational inheritance. To that aim, we generated a human A-375 melanoma cell line derivative stably expressing EGFP by lentiviral vector infection [22], then xenografted the cells into mice and finally analysed mature spermatozoa from these mice, as shown in the schematics in Fig. 1. The analysis of EGFP-expressing A-375 cell lines is shown in Fig. 2. Fig. 2A shows representative EGFP-specific RT-PCR assays using RNA extracted from whole A-375 cells after infection with EGFP-encoding lentivirus (lane 1). We also analysed the extracellular fraction sequentially purified from the culture medium following well-established protocols for exosome isolation [23]: that fraction proved indeed reactive to anti-CD81 antibody (Fig. 2C), a marker of exosomes (41), and henceforth will be referred to as exosomal fraction or preparation, or exosomes, for simplicity. EGFP RNA was found in this fraction from EGFP-infected A-375 cells (lane 5), but not in total RNA from non-infected A-375 cells (lane 3) or from their purified exosomal fraction (lane 4). Using direct PCR amplification we also detected EGFP sequences in genomic DNA from infected A-375 cells (Fig. 2B, lane 1) and, in lower abundance, in their released exosomal fraction (lane 6). EGFP sequences were instead absent from non-infected cells (lane 4) and their derived exosome preparation (lane 5). Western blot analysis using EGFP-specific antibody (Fig. 2C) revealed EGFP protein in extracts from EGFP-infected A-375 whole cells (lane 3) and released exosomes (lane 4), while extracts from either non-infected A-375 cells (lane 1) or their exosomes (lane 2) gave no signal. These data indicate that EGFP RNA and, to a lesser extent, DNA and protein, are released from tumour cells and are recovered in the extracellular purified exosomal fraction.

**Figure 3:**
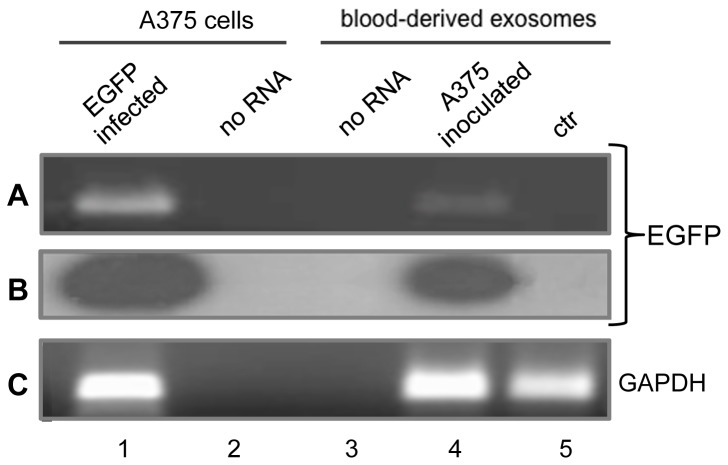
EGFP-specific RNA in circulating blood from A-375/EGFP-inoculated mice. **A:** Ethidium bromide staining of specific RT-PCR products from RNA extracted from EGFP-infected A-375 cells (lane 1) and blood-purified extracellular exosomal fraction from inoculated (lane 4) and non-inoculated (lane 5) mice. No RNA and no primers were added to the amplification mix in lanes 2 and 3, respectively. **B:** EGFP hybridization pattern. The gel in A was blotted on filter, hybridized with ^32^P-end labelled EGFP-specific probe, washed and autoradiographed. **C**: GAPDH-specific amplification products from the same samples.

We next inoculated the EGFP-expressing A-375 cells subcutaneously in athymic male mice. After 45 days of xenograft growth, we analysed the blood of the xenografted animals to assess the presence of EGFP-containing RNA – which is obviously not encoded by either the human or the murine genomes - as an unambiguous marker originating from the EGFP-engineered tumour cells. RT-PCR analyses are shown in Fig. 3. In panel A, EGFP-specific RNA molecules were amplified from RNA extracted from the exosomal fraction prepared from 4 ml of mouse plasma of inoculated (lane 4), but not of non-inoculated (lane 5), animals. The identity of the amplified RNA product was further confirmed by hybridising with an EGFP-specific probe (panel B). We also carried out parallel PCR assays omitting the RT step (Fig. S1), which confirmed that EGFP amplification products definitely originate from RNA in five independent assays. These data demonstrate that EGFP-containing RNA is released from xenografted tumour cells into the circulating blood of the animals and is associated with the exosomal fraction.

### Tumor-released EGFP RNA is taken up by epididymal spermatozoa of xenografted mice

**Figure 4:**
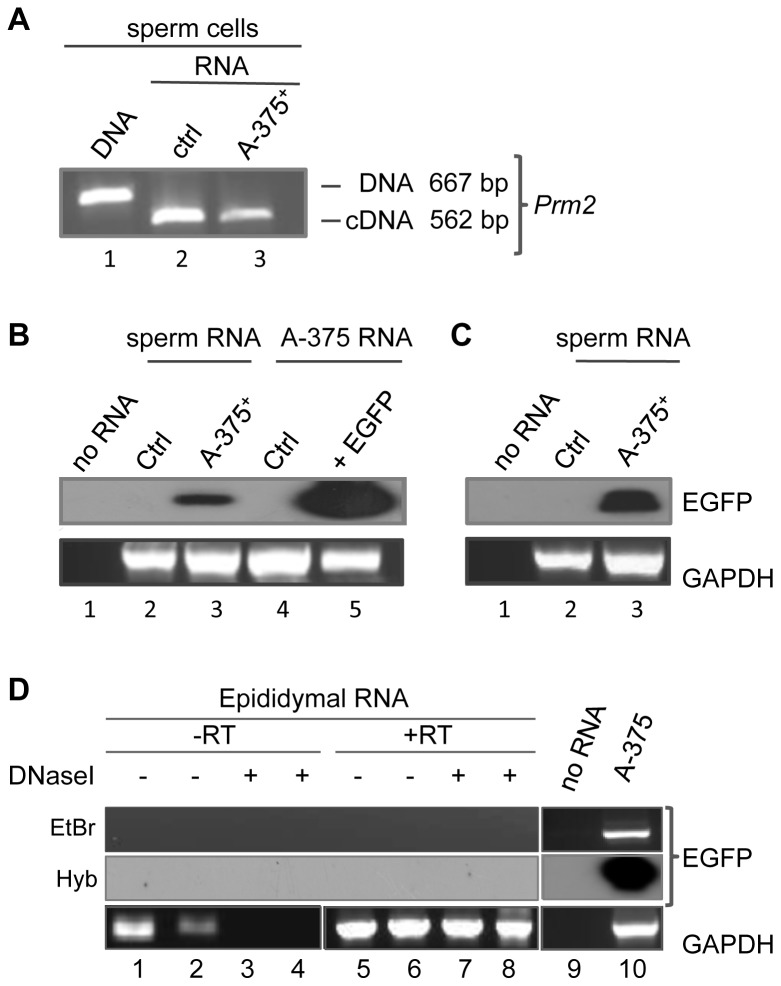
EGFP RNA is present in spermatozoa of mice inoculated with EGFP-infected A-375-cells. **A:** Murine protamine 2 gene (*Prm2*) amplification products used to select DNA-free RNA samples. Exemplifying gel of *Prm2-*specific PCR amplification products of intron-containing DNA from the mouse sperm genome (lane 1) and RT-PCR products from RNA extracted from spermatozoa of non-inoculated (lane 2) and EGFP-expressing A-375^+^ inoculated (lane 3) mice, both showing the spliced *Prm2* form. **B:** Southern blot hybridization of RT-PCR amplified RNA from**:** spermatozoa of non-inoculated control (lane 2) and A-375^+^ inoculated (lane 3) mice, and from non-infected (lane 4) and EGFP-infected (lane 5) A-375 whole cells. Hybridization was carried out with an EGFP-specific internal probe. Lane 1 is a no-RNA control. The bottom panel shows RT-PCR amplification products from the same samples using GAPDH-specific primers as a loading control. **C:** Southern blot hybridization of RT-PCR amplified RNA from spermatozoa from a control mouse (lane 2) and from a single EGFP-expressing A375^+^ inoculated mouse (lane 3); lane 1 shows a no RNA reaction. As in B, the bottom panel shows GAPDH amplification from the same samples. **D:** RT-PCR amplification with (+RT) or without (-RT) reverse transcription step of RNA extracted from sperm-depleted epididymis from two inoculated EGFP mice. No EGFP-specific amplification products were visible by ethidium bromide staining (EtBr) nor by Southern blot hybridization (Hyb) using an EGFP radioactive probe. The bottom panel shows GAPDH amplification from the same samples.

We next asked whether the EGFP RNA released from xenografted tumour cells into the blood of the recipient animals can eventually be transferred to the mouse gametes. Preparations of epididymal spermatozoa free of somatic cells were surgically obtained following established protocols [24] from both tumour cell-inoculated and non-inoculated animals. RNA was then extracted from the sperm heads prepared from both animal groups. We routinely verified that spermatozoal RNA preparations were not contaminated with genomic DNA by amplifying with a pair of oligonucleotides flanking the intron of the protamine 2 (*Prm2*) gene [25]. Figure 4A shows RT-PCR amplifications of spermatozoal RNA preparations from both non-inoculated controls (lane 2) and from A-375 cell-inoculated (lane 3) mice: RT-PCR selectively amplified a 562 bp-long spliced transcript fragment from sperm RNA, but no larger fragment, whereas a 667 bp-long intron-containing *Prm2* gene fragment was only visible by genomic DNA direct amplification (lane 1), indicating that RNA preparations are indeed DNA-free.

At this point we assessed the presence of EGFP-containing RNA sequences by RT-PCR using pairs of EGFP-specific oligonucleotides: a specific product was amplified using spermatozoal RNA extracted from mice inoculated with EGFP-expressing A-375 cells but not from control animals: specifically, we carried out five distinct experiments to assess the presence of EGFP-specific RNA in spermatozoa from mice inoculated with EGFP-expressing A-375 cells; i) a pool of spermatozoa obtained from ten mice (Fig. 4B, lane 3); ii) a pool of spermatozoa from two animals, also with positive results (not shown); iii) single spermatozoa preparations obtained from three individual mice, in one of which no EGFP signal was detected, while one of the two positives is shown in Fig. 4 C, lane 3. Parallel PCR assays omitting the RT step were carried out which confirmed that EGFP amplification products originate from RNA (Fig. S2). The RNA from the spermatozoa of non-inoculated control animals was consistently negative to EGFP-specific amplification in all experiments (Fig. 4B, lane 2 and Fig. 4C, lane 2).

A number of control experiments were designed to avoid all possible sources of contamination in all steps. Particular care was taken to avoid blood cells contaminating epididymal spermatozoa: the latter were routinely collected by “swim up” selection [24] and each sperm preparation was examined under the microscope to ensure that no somatic cells were present. Only somatic cell-free sperm preparations were used in our assays. To unambiguously rule out the possibility that rare blood cells might be the source of the EGFP RNA detected in spermatozoa, RNA was extracted from increasing aliquots (20 to 160 microliters) of whole blood from EGFP-expressing A-375 inoculated mice and amplified by RT-PCR in order to determine the minimal volume of contaminating blood that would be required to amplify EGFP RNA. Our results indicate that EGFP signal was not detected even with the highest tested volume (160 microliters), which exceeds by 50–60 fold the volume of possible inadvertent contaminant (2–3 µl) of sperm preparations (data not shown). Another potential source of sperm contamination might be represented by epididymal somatic cells. To rule out such a possibility, the epididymis from three A-375 inoculated mice and from a non-inoculated animal were squeezed, thoroughly depleted of sperm cells and used to extract RNA. RT-PCR amplification in triplicate, followed by Southern blot hybridization, indicated that no EGFP sequences are detectable in epididymis somatic cell samples (Fig. 4, panel D), under conditions under which the spermatozoal RNA from the same animals was positive for EGFP expression (as exemplified in Fig. 4C, lane 3). On these grounds, we feel that we can confidently rule out the possibility that sperm EGFP RNA is a blood- or epididymis-derived contaminant. In summary, therefore, these results demonstrate that RNA molecules, originally generated in somatic A-375 cells, are transferred to male germline cells and are present in viable epididymal spermatozoa, implying that the soma-to-germline barrier can be crossed.

## Discussion

The present experiments indicate that tumour cell-derived RNAs can be transferred to epididymal spermatozoa, unveiling a direct route connecting somatic cells to mature male gametes. Similarly, a variety of informational molecules released from tumour cells [26], [12] may in principle be delivered via this route into germ cells, with a potential to influence the transgenerational risk of cancer transmission to the progeny. It will be interesting to clarify in future work whether this occurs as a special event under stressing conditions, as is the xenotransplantation of human tumour cells in nude mouse strains, or whether it also occurs under physiological conditions.

EGFP RNA transcribed in human tumour cells is used here as a non-human and non-murine expressed tracer; we find EGFP RNA to associate with an extracellular fraction processed for exosome purification and expressing exosomal markers in every step, from tumour cells to blood and hence to sperm. Exosomes are abundantly released from tumours [19] and described as carriers of a complex repertoire of genetic information available for horizontal gene transfer [20], [27], [28]. These circulating particles can propagate towards other organs and the germline, potentially affecting the transgenerational cancer risk of the progeny [7], [29]. Exosome-like vescicles have actually been detected both in the seminal fluid [30] and in the epididymis [31], [32] of various mammals, including humans, in close contact with spermatozoa. In this context, nanoparticles are thought to act as sperm-delivering carriers of factors required for sperm maturation and fertilizing ability [33] suggesting that exosomes are good carriers capable of crossing the barrier between somatic tumour cells and sperm cells. It is worth recalling that, in spite of the highly compact structure of the sperm chromatin, sperm nuclei have empty space in which viral particles can be accommodated [34]. The finding that spermatozoa are the final recipients of tumour-released RNA suggests that the latter might propagate to the next generation at fertilization.

Work from our and other laboratories indicates that spermatozoa act as vectors not only of their own genome, but also of foreign genetic information, based on their spontaneous ability to take up exogenous DNA and RNA molecules that are then delivered to oocytes at fertilization with the ensuing generation of phenotypically modified animals [35]–[37]. In cases in which this has been thoroughly investigated, the sperm-delivered sequences have been seen to remain extrachromosomal and to be sexually transmitted to the next generation in a non-Mendelian fashion [38]. The modes of genetic information delivery in this process are closely reminiscent of those operating in RNA-mediated paramutation inheritance, whereby RNA is the determinant of inheritable epigenetic variations [16], [17]. In conclusion, this work reveals that a flow of information can be transferred from the soma to the germline, escaping the principle of the Weismann barrier [39] which postulates that somatically acquired genetic variations cannot be transferred to the germline.

## Methods

### Ethics Statement

All animal procedures were conducted under the approval of the Italian National Institute of Health. Animal care was conformed to the European Council Directive 86/609/EEC and all experiments including animals were approved by the review board of the Italian National Institute of Health (Istituto Superiore di Sanità, ISS) and authorized by the Italian Ministry of Health. Crl:CD1-Foxn1nu athymic male mice were chosen as recipients of tumorigenic huma melanoma cells. Animals were sacrificed by cervical dislocation. All efforts were made to minimize suffering.

### Cell cultures

A-375 human melanoma cell line (ATCC) were mantained in DMEM medium, supplemented with 10% fetal bovine serum (FBS), 100 U/ml streptomycin, 100 µg/ml penicillin. Cells were cultured in 5% CO2 at 37°C, collected by centrifugation and supernatants were then used for nanovesicle isolation.

### Inoculation of tumor cells in nude mice, collection of serum and sperm samples

A-375 human melanoma derivative cell lines stably expressing EGFP were generated by infection with pWPXLd constitutive lentiviral plasmid vector as described [22]. Crl:CD1-Foxn1nu male mice (Charles River) 4–5 weeks old were injected subcutaneously with 5×10^6^ human melanoma cells. Tumour growth was monitored by caliper and mice were sacrificed when tumours reached the size of about 1 cm^3^. Plasma samples were collected from 10 xenografted mice and from 10 non-inoculated control mice and processed according to established protocols for the isolation of exosomes (details below). Epididymal spermatozoa were surgically obtained from mice as previously described [40] and collected from the following animals inoculated with EGFP-expressing A375 cells: i) a pool of ten mice, ii) a pool of two mice, and iii) three individual animals; as well as from matched non-inoculated control mice.

### Exosome-containing fraction purification

Extracellular material was processed from A-375 cell supernatants according to established protocols for exosome purification [23]. Briefly, supernatants were centrifuged for 5 min at 300 g, 20 min at 1,200 g and 30 min at 10,000 g to remove cell debris, then filtered through a 0.22 µm filter (Millipore Corp., Bedford, MA). Ultracentrifugation of the extracellular filtrate at 100,000 g for 60 min at 4°C using a Sorvall WX Ultra Series centrifuge in a F50L-24×1.5 rotor (Thermo Scientific) yields the sedimentation of a fraction expected to contain exosomes (23) and indeed reactive to anti-CD81 antibody, a marker of exosomes and nanovesicles (41). The resulting pellet (indicated as exosomes, or exosomal preparation or fraction) was washed in a large volume of PBS and again ultracentrifuged at 100,000 g for 60 min. The exosomal preparation was either resuspended in PBS or dissolved in lysis buffer/Trizol for further analyses. Exosome preparations were obtained from plasma following a slightly different protocol [41]. Mouse plasma was separated from total blood by centrifuging at 2,000 rpm for 20 min. The supernatant (routinely, 2 to 6 ml of separated plasma was used) was sequentially centrifuged for 30 min at 500 g and for 45 min at 12,000 g, filtered through a 0.22 µm Millipore filter and ultracentrifuged at 110,000 g for 90 min at 4°C. The resulting pellet was washed in PBS and collected by ultracentrifugation at 110,000 g for 1 h.

### DNA and RNA extraction

DNA was extracted from exosome preparations and from whole cells in lysis buffer (50 mM TRIS-HCl pH 7, 2 mM EDTA pH 8, 1% SDS), incubated overnight at 37°C with 50 µg/ml Proteinase K and 145 µg/ml RNAse A, followed by several phenol/chlorophorm extractions. DNA samples were ethanol precipitated and resuspended in sterile water for further analysis. Cultured cells, extracellular exosome preparations and spermatozoa were individually pooled and lysed in Trizol reagent (Life Technologies, Invitrogen); total RNA was extracted according to manufacturer’s protocols. Extraction from sperm was slightly modified, in that sperm pellet was resuspended in Trizol and homogenized in a teflon-glass homogenizer prior to continue with the RNA isolation procedure.

### PCR and RT-PCR

Total RNA (1 µg) was reverse transcribed into cDNA using the SuperScript Reverse Transcriptase kit (Life Technologies, Invitrogen) and random hexamers as primers. 2 µl of cDNA were PCR amplified with primer pairs specific for EGFP (Forward 5′-TCTATATCATGGCCGACAAGC-3′, Reverse 5′-GGTGTTCTGCTGGTAGTGG-3′) and GAPDH (Forward 5′-ATTCAACGGCACAGTCAAGG-3′, Reverse 5′-AAGGTGGAAGAGTGGGAGTT-3′) transcripts, these latter do not span introns. The integrity and purity of RNA extracted from sperms were assessed by a RT-PCR based assay [25] using specific, exon boundary primers for Prm2 mRNA (Forward 5′-CTTGGGCAGGTGACTATTCC-3′, Reverse 5′-CTCCTCCTCCAATCCAGGTC-3′).

The amplification products were fractionated through 1.5% agarose gels, then transferred to a nylon membrane (Hybond N+, GE Healthcare) in 0.4M NaOH. The internal probe (5′-TGCACGCTGCCGTCCTCGAT-3′) was ^32^P end-labelled using T4 polynucleotide kinase (Life Technologies, Invitrogen). Filters were washed for 10 min at room T° in 2xSSC and prehybridised in HB (20 mM NaP; 0.1% NaPP; 1M NaCl; 1% SDS) at 42°C for 2 hours. The labelled probe was denatured at 95°C for 5 min and mixed with HB; the hybridization was carried out at 42°C for 16–20 hours. Filters were washed twice in 2x SSC and 0.2% SDS for 15 min at 25°C, twice in 0.5x SSC and 0.1% SDS for 15 min at 42°C, and twice in 0.2x SSC and 0.1% SDS for 15 min at 42°C, then exposed to X-ray film.

### Western immunoblotting assays

Purified exosome preparations, or whole cells, were lysed in lysis buffer (1% Triton X-100, 0.1% SDS, 0.1 M Tris HCl, pH 7.0) or in AKT buffer (20 mM Tris-HCl pH 7.4, 150 mM NaCl, 10% glycerol, 1% NP40) respectively, both containing 10 µg/mL aprotinin and 2 mM phenylmethylsulfonyl fluoride protease inhibitors (Hoffman-La Roche). Protein concentration was assessed using the Bradford assay (Biorad Laboratories). Protein aliquots were diluted in Laemmli loading buffer, analyzed by SDS-PAGE and electroblotted on nitrocellulose membranes (PROTRAN Whatman GmbH). Membranes were incubated with rabbit anti-EGFP (AbCam) and mouse anti-CD81 (B-11, Santa Cruz) primary antibodies, then with HRP-conjugated anti-rabbit or anti-mouse secondary antibodies (Amersham Biosciences). Signals were detected by enhanced chemoluminescence (Pierce).

## Supporting Information

Figure S1 **EGFP amplification products predominantly derive from RNA in circulating plasma exosomes.** PCR amplification assays were carried out without RT (-RT) using RNA extracted from the plasma of two distinct pools (identified as 1 and 2) of mice inoculated with A-375 cells (A-375^+^) and from one pool of non-inoculated control animals (ctrl pool). **A**: EGFP amplification assays (in triplicate) hybridized with ^32^P-labelled EGFP probe; in the absence of RT no EGFP signal is detected in samples from either A-375-inoculated (lanes 1–6) or non-inoculated (lanes 7–9) mice. **B**: Ethidium bromide staining of GAPDH DNA amplification products: the same samples were tested for the presence of contaminating DNA, which is present in samples from pool 1 (lanes 1–3) and Ctrl (lanes 7–9), but not from pool 2, yet all failed to yield EGFP products in the no-RT control in A.
(TIF)

Figure S2 **EGFP amplification products in sperm cells predominantly derive from RNA.** PCR amplification assays were carried out without RT (-RT) using RNA from spermatozoa from three A-375-xenografted (A-375^+^ a, b, c) and one non-inoculated (ctrl) mice. **A**: Southern blot hybridization (^32^P-labelled EGFP probe) reveals no EGFP amplification product from sperm RNA (lanes 1–4), or from negative control reaction (lane 5); a positive control is shown in lane 6. **B**: Ethidium bromide staining of GAPDH DNA amplification products.
(TIF)
