# Supplementary figures and images for: Soma-to-Germline Transmission of RNA in Mice Xenografted with Human Tumour Cells: Possible Transport by Exosomes
Source: PLoS One. 2014 Jul 3;9(7):e101629. doi: 10.1371/journal.pone.0101629 (PMC4081593; doi:10.1371/journal.pone.0101629)

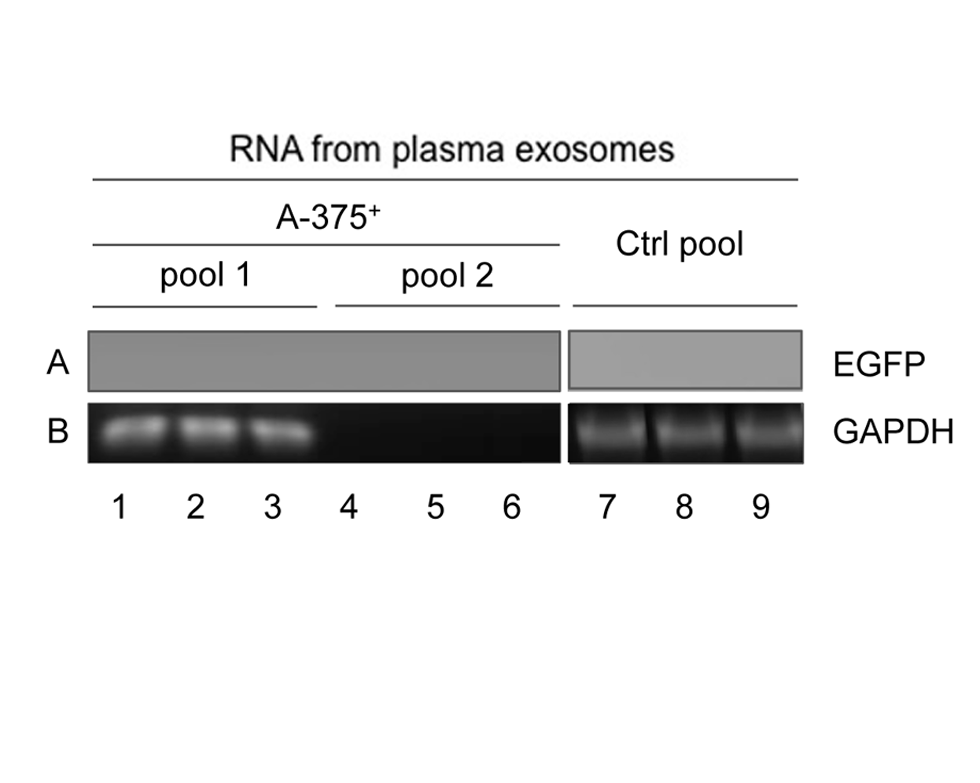

Supplement: Figure S1 — EGFP amplification products predominantly derive from RNA in circulating plasma exosomes. PCR amplification assays were carried out without RT (-RT) using RNA extracted from the plasma of two distinct pools (identified as 1 and 2) of mice inoculated with A-375 cells (A-375+) and from one pool of non-inoculated control animals (ctrl pool). A: EGFP amplification assays (in triplicate) hybridized with 32P-labelled EGFP probe; in the absence of RT no EGFP signal is detected in samples from either A-375-inoculated (lanes 1–6) or non-inoculated (lanes 7–9) mice. B: Ethidium bromide staining of GAPDH DNA amplification products: the same samples were tested for the presence of contaminating DNA, which is present in samples from pool 1 (lanes 1–3) and Ctrl (lanes 7–9), but not from pool 2, yet all failed to yield EGFP products in the no-RT control in A. (TIF) [file pone.0101629.s001.tif]

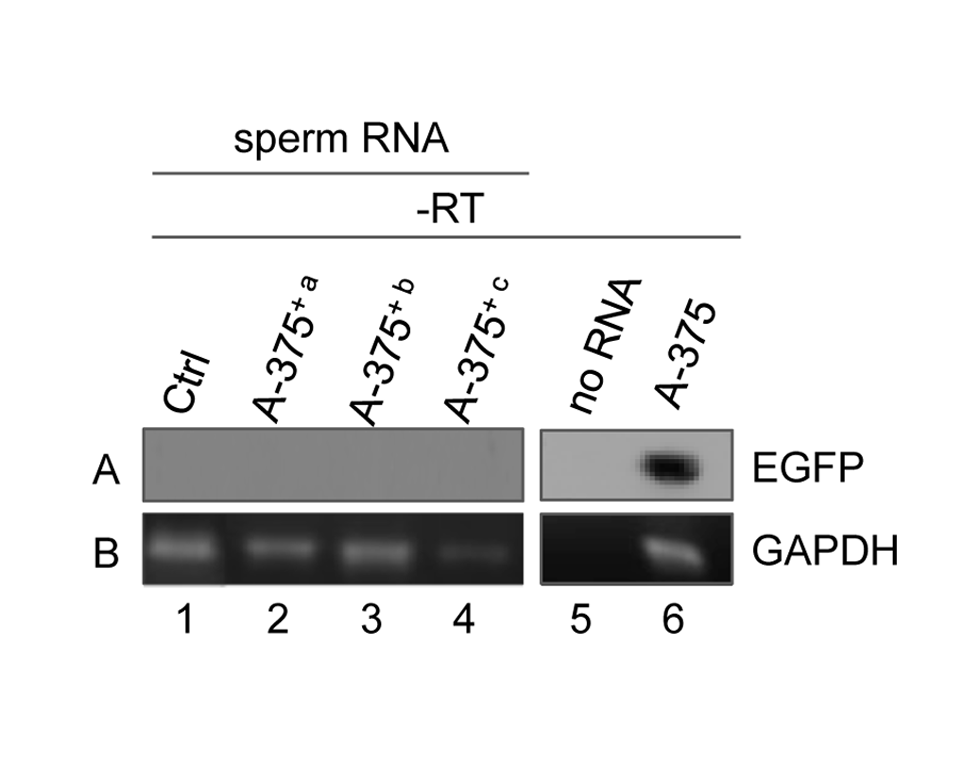

Supplement: Figure S2 — EGFP amplification products in sperm cells predominantly derive from RNA. PCR amplification assays were carried out without RT (-RT) using RNA from spermatozoa from three A-375-xenografted (A-375+ a, b, c) and one non-inoculated (ctrl) mice. A: Southern blot hybridization (32P-labelled EGFP probe) reveals no EGFP amplification product from sperm RNA (lanes 1–4), or from negative control reaction (lane 5); a positive control is shown in lane 6. B: Ethidium bromide staining of GAPDH DNA amplification products. (TIF) [file pone.0101629.s002.tif]
